# Surgical Treatment of Solitary Enchondromas of the Hand

**DOI:** 10.7759/cureus.7497

**Published:** 2020-04-01

**Authors:** Sercan Çapkin, Ali Cavit, Kutay Yilmaz, Tufan Kaleli

**Affiliations:** 1 Orthopaedics and Traumatology, Faculty of Medicine, Aksaray Education and Research Hospital, Aksaray University, Aksaray, TUR; 2 Orthopaedics, Uludag University School of Medicine, Bursa, TUR; 3 Orthopaedics, Faculty of Medicine, Uludag University, Bursa, TUR

**Keywords:** hand, curettage, autologous, bone graft, enchondroma, bone tumor, recurrence

## Abstract

Objective: The present retrospective study evaluated the clinical and radiologic results of patients who underwent complete curettage and autologous bone grafting for hand-located isolated enchondromas with a minimum follow-up period of one year.

Patients and Methods: Thirty-two patients with a follow-up period of at least 12 months who underwent operation between August 2010 and October 2018 due to the presence of solitary enchondroma of the hand were included in the study. All patients underwent complete curettage and filling of the defect via autologous bone grafting. Autologous bone graft was harvested from the iliac crest and distal radius in 24 and eight patients, respectively. The patients underwent radiography on the first postoperative visit and at six weeks, 12 weeks, and annually. The range of movement of the finger joint was evaluated by comparing it with the healthy contralateral side. Functional outcomes and radiologic outcomes were evaluated. The frequency of complications and recurrences were established.

Results: Twelve patients were male and 20 were female. The average age was 34 (range: 16-56) years. The most common digit involved was the little finger (nine cases, 28.125%); the proximal phalanx was the most common location (17 cases, 53.125%). Control radiography in the sixth week revealed graft consolidation in all patients. No case of nonunion or recurrence was detected clinically or radiologically, with a mean follow-up period of 54 (range: 12-96) months. Functional outcomes were classified as excellent in 28 patients and as good in four patients. The final radiographic appearances included Tordai’s group 1 in 28 bones and group 2 in four bones.

Conclusion: Curettage and autologous bone grafting are safe, costless, and effective treatment options for hand enchondroma, with satisfactory functional and radiographic outcomes. Harvesting bone graft from the distal radius provides a shorter length of hospital stay and lower complication rates compared to obtaining the graft from the iliac crest.

## Introduction

Enchondromas are benign cartilaginous tumors comprising mature hyaline cartilage and are the most common primary osseous tumors of the hand [1-6]. They are often solitary lesions in the phalanges. The malignant transformation of a solitary enchondroma into chondrosarcoma is exceedingly rare [7]. However, they may rarely present as multiple lesions. Multiple enchondromas, known as Ollier disease (enchondromatosis) and Maffucci syndrome (a combination of multiple enchondromas and hemangiomas) have a risk of malignant transformation, with the risk increasing up to 25% in patients with Ollier disease and up to 100% in those with Maffucci syndrome [8].

Enchondromas generally develop in the third and fourth decades of life and have a predilection for the ulnar-sided tubular bones of the hand [9]. Proximal phalanges are the most frequently affected part of the hand, followed by the middle phalanges, metacarpals, and rarely distal phalanges [9-10].

Enchondromas are slow-growing tumors and generally asymptomatic. Diagnosis is often made via incidental radiological imaging. The most common presenting symptoms of enchondromas include pain, swelling, and deformity. However, some enchondromas are diagnosed with the initial presentation of pain after minor trauma, which results in a pathological fracture caused by the expansile remodeling of the lesion. In most series, pathologic fractures are observed in 40%-60% of patients at initial presentation [11-12].

Symptomatic enchondromas are generally treated via surgery. The aim of surgical treatment is to confirm the histologic diagnosis and to prevent the development of pathologic fractures and deformity. Complete curettage and filling of the defect via autologous bone grafting have been the conventional methods for the surgical treatment of hand enchondromas [13]. The present retrospective study evaluated the clinical and radiologic results of patients who underwent complete curettage and autologous bone grafting for hand-located isolated enchondromas with a minimum follow-up period of one year.

## Materials and methods

This retrospective study was approved by the Clinical Research Ethics Committee. The study was designed to retrospectively evaluate the medical records of 41 patients with enchondroma of the hand between August 2010 and October 2018. The exclusion criteria were patients with Ollier disease or Maffucci syndrome as well as those with inadequate follow-up. Four patients with Ollier disease, one patient with Mafucci syndrome, and four patients who had a follow-up period of less than one year were excluded. Thirty-two patients with a follow-up period of at least 12 months who underwent operation due to the presence of solitary enchondroma of the hand were included in the study. All patients underwent complete curettage and filling of the defect via autologous bone grafting. In 24 patients, an autologous bone graft was harvested from the iliac crest under general anesthesia. For the remaining eight patients, an autologous bone graft was harvested from the ipsilateral distal radius bone under regional anesthesia. Informed consent was preoperatively requested from all patients for the harvesting of the graft from either the iliac crest or the distal radius bone.

**Figure 1:**
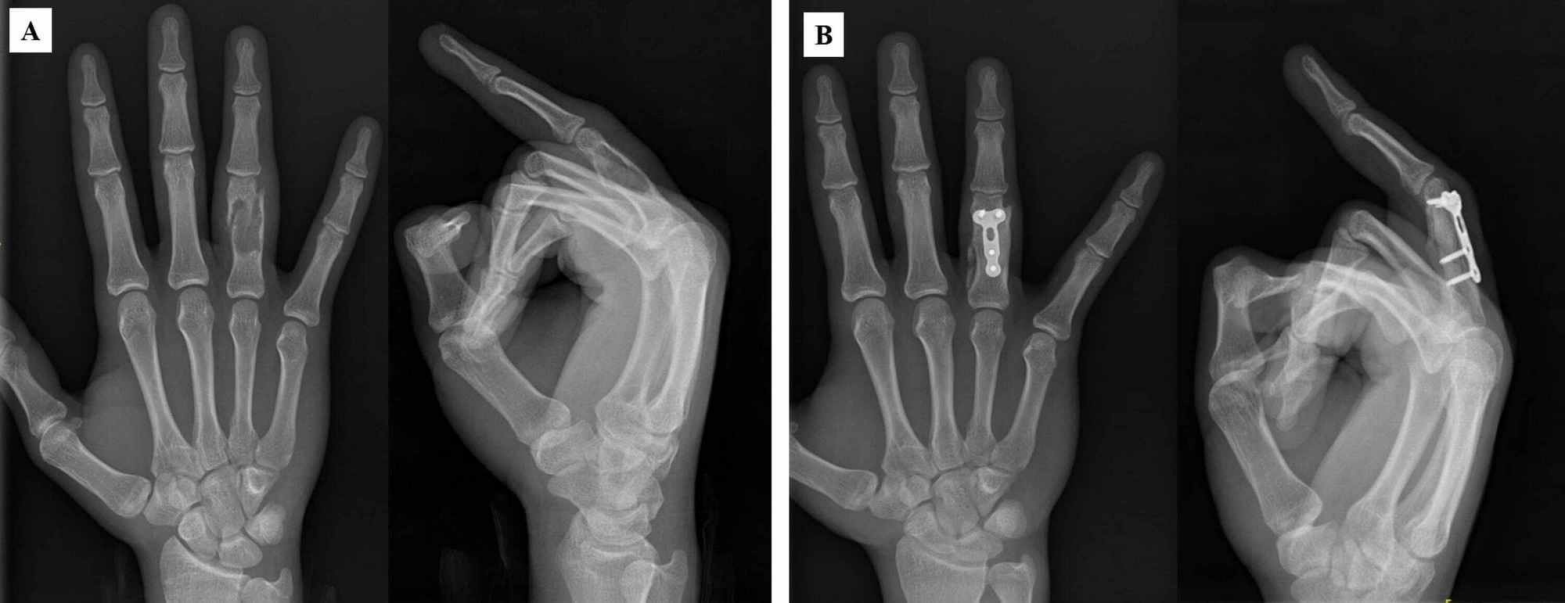
A: A 17-year-old patient with displaced proximal phalangeal pathological fracture. B: Curettage of the enchondroma and filling the defect with autologous bone graft from iliac crest, and plate fixation (postoperatively sixth month, bone graft was well incorporated).

Eight patients had a pathologic fracture at the initial presentation. One patient required primary open reduction and internal fixation of a displaced proximal phalangeal pathologic fracture, followed by curettage of the enchondroma and bone grafting (Figure 1). All other patients underwent surgery after the fracture had healed via conservative treatment. The average duration from the fracture to final surgery was eight (4-12) months. The tumoral tissue was removed from all patients and sent for histologic examination. The diagnosis of enchondroma was histologically proven in all patients.

After the surgery, all patients wore a short-arm splint for three weeks in the intrinsic plus position. After removing the splint, patients underwent passive and active exercises. Patients who received grafts from the iliac crest were hospitalized for one day, whereas those who received grafts from the distal radius were discharged on the same day.

The patients underwent radiography on the first postoperative visit and at six weeks, 12 weeks, and annually. The results presented here are based on each patient's most recent follow-up. The range of movement of the finger joint was evaluated by comparing it with the healthy contralateral side. Functional outcomes were determined according to the four criteria described by Takigawa [14]: 1 = appearance (acceptable or not), 2 = the patients’ active range of motion (≥80% in comparison with the contralateral side), 3 = the patients’ grip strength (≥80% in comparison with the contralateral side), and 4 = radiographic evidence of healing without shortening, deformity, osteoarthrosis, or tumor recurrence. The results were graded as excellent (criteria 4), good (criteria 3), fair (criteria 2), and poor (criterion 1 or 0). Radiologic outcomes were measured via X-ray imaging using the classification system described by Tordai [11]: group 1, normal cortical and cancellous bone or presence of a bone defect of less than 3 mm in diameter; group 2, bone defect of 4-10 mm in diameter with no clear-cut recurrence; and group 3, bone defect larger than 10 mm with the characteristics of an enchondroma. The average follow-up duration was 54 (range 12-96) months. Furthermore, we established the frequency of complications and recurrences.

## Results

Twelve patients were male and 20 were female. The average age was 34 (range: 16-56) years. In 12 patients, the left hand was affected whereas in 20 patients, the right hand was affected. The age distribution showed predisposition in the third and fourth decade of life. The distribution of enchondromas in the hand is summarized in Table 1. The most common digit involved was the little finger (nine cases, 28.125%); the proximal phalanx was the most common location (17 cases, 53.125%). No enchondromas were found in the carpal bones. With regard to initial presentation, 16 (50%) patients presented with pain or swelling, eight (25%) with pathological fractures after a variety of trauma, and eight (25%) with incidental findings after the investigation of vague hand pain.

**Table 1:** Distribution of enchondromas in the hand.

	Distal phalanx	Middle phalanx	Proximal phalanx	Metacarpal	Total
Thumb	1	0	1	1	3
Index	0	2	5	1	8
Long	1	0	4	0	5
Ring	0	2	4	1	7
Little	1	2	3	3	9
Total	3	6	17	6	32

**Figure 2:**
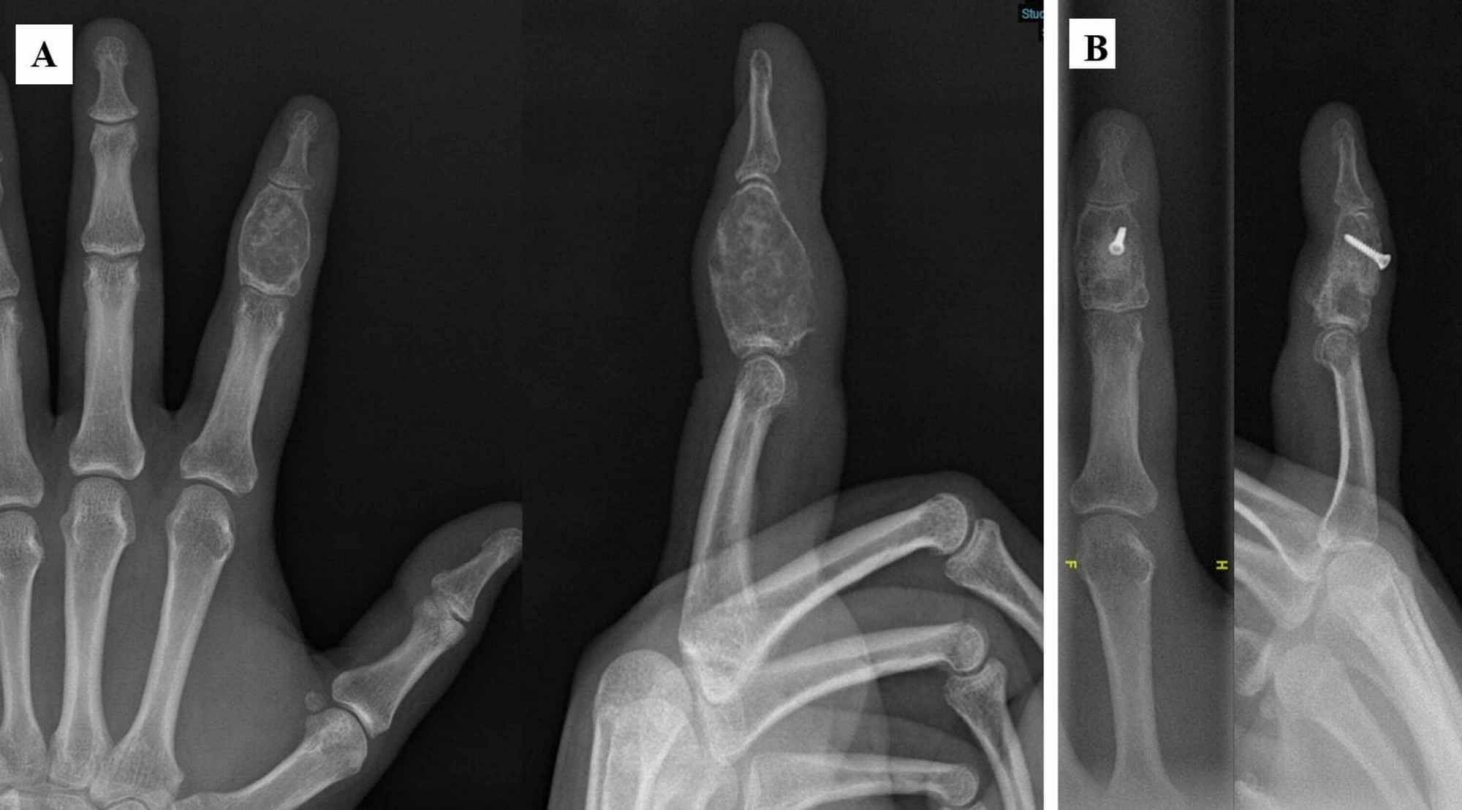
A: 32-year-old patient with giant enchondroma at the middle phalanx. B: Bone graft from iliac crest was fixed with a screw and was well incorporated at 12th month postoperatively.

There were no major complications, such as preoperative neurovascular or tendon injuries and postoperative fracture, observed in any of the patients. Postoperative finger joint stiffness was detected in five patients at the final follow-up. Four out of these five patients already had preoperative stiffness; three out of these five patients had an active range of motion (<80%) in comparison with the contralateral side and the other two patients had an active range of motion (>80%) in comparison with the contralateral side. A second surgery, such as tenolysis, was not performed in any patient as they had grip strengths of ≥80% in comparison with the contralateral side and did not accept a second operation. One patient complained of swelling in the middle phalanx at the 12-month postoperative follow-up; however, this patient had preoperative swelling (giant enchondroma, Figure 2).

Three patients (bone graft obtained from the iliac crest) complained about donor site pain or related thigh numbness. Wound infection was observed in two patients (bone graft obtained by iliac crest), which was successfully treated with a course of antibiotics and daily dressing.

Control radiography in the sixth week revealed graft consolidation in all patients. No case of nonunion or recurrence was detected clinically or radiologically, with a mean follow-up period of 54 months. At the final follow-up, 26 patients were symptom-free. Functional outcomes were classified as excellent in 28 patients and as good in four patients. No patient was classified as fair or poor. In three patients, the result was assessed as good on account of restricted mobility caused by postoperative finger stiffness. The other patient was assessed as good due to an unacceptable finger appearance. The final radiographic appearances included Tordai’s group 1 in 28 bones and group 2 in four bones. No bone was classified as group 3.

## Discussion

The findings of our study are largely consistent with those of previous studies [1-4, 15-17]. In most patients, enchondromas were diagnosed in the third and fourth decades of life. Enchondromas were most commonly located in the little finger (28.125%) and proximal phalanx (53.125%).

The treatment methods for enchondromas include conservative regular follow-up or surgical excision via curettage [13, 18]. Whether the surgical removal of solitary enchondromas is necessary remains controversial [1]. However, this method provides histologic diagnosis, eliminates clinical symptoms, and ensures mechanical stability of the bone by minimizing the risk of pathologic fractures. In this context, the aim of the surgical treatment is to confirm histologic diagnosis, prevent malignant degeneration, and reduce the risk of deformity and pathological fracture that may occur after tumor tissue growth [1,13,18]. There are several surgical treatments for enchondroma, including curettage alone, curettage and autogenous bone grafting, and curettage and artificial bone grafting or cement injection [5,19-22]. Curettage alone leaves dead spaces and weakens the bone, which may increase the risk of fracture [20].

A study in which the necessity of cancellous bone grafting was investigated found that additional bone grafting for the treatment of enchondromas is not necessary and that this procedure should be reserved for particular indications. The study concluded that bone grafting was necessary only if the cortical bone was thin, was at a high risk of fracture, or if the lesion was located close to an articular surface [19]. In another study where 102 enchondromas were operated using various techniques, it was suggested that surgeries should be performed with an allograft or without any cavity fill to avoid the problem of donor site morbidity. The authors found that an autologous graft did not influence the healing process; it did not reduce healing time, recurrence, and complications or increase the range of motion [10]. Another study compared an artificial bone substitute with an iliac crest bone graft to evaluate the treatment outcomes of enchondroma of the hand. The authors did not find any differences between the groups upon functional evaluation. However, they observed that artificial bone substitutes took twice as long to achieve bone integration. Nevertheless, they favored the use of bone substitutes because it eliminated the problem of donor site morbidity, reduced local anesthetic use and surgical time, and permitted outpatient treatment [23].

Curettage followed by the application of an iliac crest or distal radius autograft was the traditional treatment for hand enchondroma at our hospital. The main advantage of autologous bone grafting is that it provides osteoconductive, osteoinductive, and osteogenic properties. Moreover, the average consolidation time is shorter than that in patients who undergo allografting. In our study, graft consolidation was detected in all patients during control radiography in the sixth week. In the literature, it was reported that incorporation developed within a similar period among patients with autografts [24-25]. Our study demonstrated that usually good or excellent functional and radiologic results were obtained from curettage and autologous bone grafting with a histological diagnosis of enchondroma.

Approximately 20% of patients experienced complications such as persistent donor site pain, nerve injury, hematoma formation, infection, incisional hernia, and fracture after harvesting bone graft from iliac crest. In particular, gait derangement was observed in patients with iliac crest bone graft harvested using the conventional technique, with an approximately 5-cm skin incision [26]. In a recent study, 48 patients with an enchondroma located in the hand were treated with an autologous bone graft, which was harvested from the iliac bone crest via a minimally invasive technique (using a Craig biopsy needle). The authors did not find any complications in the donor site [16]. In our study, minor complications, such as donor site pain or related thigh numbness and superficial infection, were observed in five patients (20,83%), which were related to the donor site (iliac crest bone graft harvested using the conventional technique).

In our study, the use of an autologous bone graft harvested from the distal radius bone decreased the complication ratio as well as the duration of hospitalization when compared with that harvested from the iliac crest bone. We preferred to use an autograft harvested from the distal radius bone in patients with a tumor size of approximately <1 cm² and female patients with cosmetic concerns. These patients were operated under regional anesthesia and were discharged on the same day. In addition, donor site pain in the iliac crest or related thigh numbness and wound infection were not observed in these patients who had grafts harvested from the distal radius bone.

In a study investigating possible factors, including sex, age, pathological fracture, location of the lesion, and preoperative stiffness affecting outcomes of surgery for enchondromas of the hand found that only preoperative stiffness was a statistically significant risk factor contributing to postoperative stiffness and occurrence of a secondary operation [27]. In our study, postoperative finger joint stiffness was detected in five patients at the final follow-up. Four out of these five patients already had preoperative stiffness.

The reported recurrence rates of enchondromas vary widely in the literature (2%-15%), with the largest published series reporting a recurrence rate of 4.5% [1, 6]. Gaulke and Suppelna reported a recurrence rate of 14% at a long-term follow-up of 21 patients [2]. All three of the recurrences were asymptomatic and were found more than 10 years after surgery. These authors reported that because regular radiologic examinations did not occur during follow-up, it was hard to decide whether these ﬁndings demonstrated late recurrences or slowly growing, asymptomatic recurrences that appeared shortly after surgery. They recommend periodic radiologic re-examination for asymptomatic recurrences before the weakness of the bone leads to a pathologic fracture [2]. The actual recurrence rate may, therefore, be higher than that reported and highlights our preference for long-term follow-up of patients. O’Connor and Bancroft suggested that recurrence may indicate malignancy and that careful review of pathology is warranted [6]. In our study, during the follow-up periods (average: 54 months), no local recurrence was observed in any patient in X-ray controls.

The limitations of this study are that it was retrospective in nature, and there were a small number of patients and short follow-up periods. In addition, there was an absence of another group such as nonsurgical treatment or artificial bone graft for comparison.

## Conclusions

Curettage and autologous bone grafting are safe, costless, and effective treatment options for hand enchondroma, with satisfactory functional and radiographic outcomes. Harvesting bone graft from the distal radius provides a shorter length of hospital stay and lower complication rates compared to obtaining the graft from the iliac crest.
